# Musculoskeletal symptoms and computer use among Finnish adolescents - pain intensity and inconvenience to everyday life: a cross-sectional study

**DOI:** 10.1186/1471-2474-13-41

**Published:** 2012-03-22

**Authors:** Paula T Hakala, Lea A Saarni, Raija-Leena Punamäki, Marjut A Wallenius, Clas-Håkan Nygård, Arja H Rimpelä

**Affiliations:** 1 School of Health Sciences, FIN-33014 University of Tampere, Finland; 2 Tampere University Hospital, PO Box 2000, FIN-33421 Tampere, Finland; 3 Tampere University of Applied Sciences, Kuntokatu 3, 33520 Tampere, Finland; 4 School of Humanities and Social Sciences, FIN-33014 University of Tampere, Finland; 5 Helsinki Collegium for Advanced Studies, FIN-00014 University of Helsinki, Finland

## Abstract

**Background:**

Musculoskeletal symptoms among adolescents are related to the time spent using a computer, but little is known about the seriousness of the symptoms or how much they affect everyday life. The purpose of the present study was to examine the intensity of musculoskeletal pain and level of inconvenience to everyday life, in relation to time spent using a computer.

**Methods:**

In a survey, 436 school children (12 to 13 and 15 to 16 years of age), answered a questionnaire on musculoskeletal and computer-associated musculoskeletal symptoms in neck-shoulder, low back, head, eyes, hands, and fingers or wrists. Pain intensity (computer-associated symptoms) and inconvenience to everyday life (musculoskeletal symptoms) were measured using a visual analogue scale. Based on the frequency and intensity, three categories were formed to classify pain at each anatomic site: none, mild, and moderate/severe. The association with time spent using the computer was analyzed by multinomial logistic regression.

**Results:**

Moderate/severe pain intensity was most often reported in the neck-shoulders (21%); head (20%); and eyes (14%); and moderate/severe inconvenience to everyday life was most often reported due to head (29%), neck-shoulders (21%), and low back (16%) pain. Compared with those using the computer less than 3.6 hours/week, computer use of ≥ 14 hours/week, was associated with moderate/severe increase in computer-associated musculoskeletal pain at all anatomic sites (odds ratio [OR] = 2.9-4.4), and moderate/severe inconvenience to everyday life due to low back (OR = 2.5) and head (OR = 2.0) pain.

**Conclusions:**

Musculoskeletal symptoms causing moderate/severe pain and inconvenience to everyday life are common among adolescent computer users. Daily computer use of 2 hours or more increases the risk for pain at most anatomic sites.

## Background

Information and communication technology (ICT) has become an important part of the lives of adolescents, the majority of whom regularly use computers for surfing the Internet, chatting, and playing games. At the same time, the prevalence of neck-shoulder and low back pain has increased among adolescents [1]. Studies among adolescents confirm a connection between musculoskeletal symptoms and the use of ICT, especially computers. Headache [2-4], neck-shoulder pain and low back pain [4-7] are more common among computer users than non-users. The risk of developing musculoskeletal pain increases with an increase in the amount of time spent on the computer [5]. Moreover, computer users' agree that computer use causes these symptoms. The findings of several studies indicate that computer use induces pain and discomfort not only in the neck-shoulder and back regions, but also in the hands, fingers, wrists, eyes, and head [8-11]. Earlier studies focused mainly on examining the relationship between pain and computer use, but there is no information on the seriousness of these symptoms and how much these symptoms affect the everyday lives of adolescents.

When assessing children and adolescents, the visual analogue scale (VAS) [12] is often used to quantify the intensity of musculoskeletal pain [13-17]. Very little is known, however, about the intensity of pain due to computer use. Konijnenberg et al. [18] described and quantified impairment due to chronic pain from an unknown cause in a study with 149 children (mean age 11.8 years). They reported that children report impaired functioning in multiple domains of daily life due to musculoskeletal pain. Roth-Isigkeit et al. [19] investigated the prevalence and characteristics of pain (e.g. pain intensity) among 735 children and adolescents (aged 10-18 years). Girls reported pain that was significantly more severe than did boys, and half of the sample reported pain lasting longer than 3 months. Among young computer users, Breen et al. [20] investigated discomfort and posture while using computers in a small sample of 68 schoolchildren (mean age 9.5 years), finding that 16% of the children reported pain, mostly in the neck or back region, at the beginning and end of a computer session. Pain intensity increased during the session [20].

In the present study, we aimed to evaluate the intensity of computer-associated musculoskeletal pain and the level of inconvenience to everyday life caused by musculoskeletal symptoms among adolescent computer users.

## Methods

### Subjects

This study was part of a longitudinal classroom study to evaluate the association between ICT use, physical and mental stress, and strain, and development in school-aged children. The sample size was 689 children from 7 schools in a major city in Finland (5 elementary and 2 middle schools) in 2004. The follow-up survey in 2006 was performed in the same schools and classes as the baseline survey. In the present cross-sectional study, 436 respondents participated including 6^th ^graders aged 12 to 13 years (n = 164, 37.6%) and 9^th ^graders aged 15 to 16 years (n = 272, 62.4%). More than half of the participants (53.7%) were girls.

### Instruments

Computer use was measured based on the response to the following open question: "How many hours per week do you usually use a computer?" The responses were categorized according to the sample distribution into three groups as follows: (i) 3.5 hours or less per week (corresponding ≤1/2 hour/day, N = 105); (ii) 3.6-13.99 hours per week (corresponding < 2 hours/day, N = 164); and (iii) 14 hours or more per week (corresponding ≥2 hours/day, N = 148). Those who did not use computers at all (N = 19) were excluded from the analysis.

The questionnaire included questions about two kinds of pain: musculoskeletal pain and computer-associated musculoskeletal pain. The latter question assessed the participants' own perception whether computer use had caused pain to them. Musculoskeletal pain was assessed with the question: "During the past half year, have you had some of the following symptoms and how often?" After the question six symptoms were mentioned (neck-shoulder, low back, head, eyes, hands, and fingers or wrists) and for each of them the following alternatives were given: a) seldom or not at all, b) about once a month, c) about once a week, and d) almost daily. Participants were asked to evaluate the level of inconvenience to everyday life due to pain using the VAS [12]. The 100-mm vertical VAS scale was marked at one end as "not inconvenient at all" and at the other end as "very inconvenient indeed". The level of the inconvenience caused by pain was assessed using the following question: "If you have musculoskeletal symptoms, how much inconvenience do they cause you in your everyday life?" The respondents were asked to make a mark on the line to indicate the level of inconvenience at the pain sites. The means for inconvenience were: head 20.7 mm (range 0-90 mm); neck or shoulders 16.1 (0-91) mm; low back 12.7 (0-76) mm; eyes 7.6 (0-77) mm; and hands, fingers and/or wrists 6.7 (0-68) mm.

Computer-associated musculoskeletal pain was assessed with the question: "Using a computer may cause symptoms (pain, aches, discomfort) in the following anatomic locations in the body. Have you experienced such symptoms?" The symptoms in neck-shoulder, low back, head, eyes, hands, and fingers or wrists, were named with the response alternatives a) not at all, b) about once a month, c) about once a week, and d) almost daily. Participants were asked to evaluate the pain intensity on the VAS-scale by the question: "If you have had computer-associated symptoms, how severe have they been?", and to make a mark on the line to indicate the intensity of each symptom. Here the endpoints were "no pain at all" and "very severe pain". The means of pain intensity were: head 15.3 mm (range 0-99 mm); neck or shoulders 15.1 (0-88) mm; eyes 10.0 (0-100) mm; low back 10.0 (0-85) mm; and hands, fingers, and/or wrists 5.3 (0-71) mm.

The VAS scores for pain intensity and level of inconvenience were each stratified into 4 groups. For pain intensity, VAS scores less than 5 mm were recorded as 0 (no pain), according to Hunfeld et al., who consider only a score of 5 mm or above to indicate the presence of pain [15,21,22]. For pain intensity and level of inconvenience symptom groups, scores of 51 mm or more were considered to indicate severe pain or severe inconvenience to everyday life; 26 to 50 mm indicated moderate pain or moderate inconvenience to everyday life; and 5 to 25 mm indicated mild pain or mild inconvenience to everyday life, respectively. Because of the small number of cases with severe pain and severe inconvenience to everyday life, the categories "severe" and "moderate" were combined into one variable "moderate/severe pain" and "moderate/severe inconvenience to everyday life".

New variables were formed by combining groups according to pain occurrence and pain intensity or level of inconvenience, as follows: "intensity of computer-associated pain" for computer-associated pain and their VAS categories, and "level of inconvenience to everyday life caused by musculoskeletal pain" for musculoskeletal pain and their VAS categories. These alternative groups were: a) seldom/no pain, no inconvenience to everyday life, b) mild pain, mild inconvenience to everyday life, and c) moderate/severe pain, moderate/severe inconvenience to everyday life. Those who reported having no or rare pain, but who evaluated their pain as mild, were classified as category b.

### Procedure

The Ethics Committee of Pirkanmaa Hospital District approved the study (Code Nr. R04013). Permission was also obtained from the school principals. An information meeting was held separately at each school for each participating class, during which the purpose of the study was explained and an information letter was delivered both to the schoolchildren and their parents. Written consent to participate was obtained from all children and their parents/guardians. The participants completed questionnaires during school hours in the spring 2006, guided by the authors and research assistants.

### Statistical analysis

Data were analyzed using SPSS for Windows, version 15.0. In the multinomial logistic regression model, variables "intensity of computer-associated pain" and "level of inconvenience to everyday life caused by musculoskeletal pain" were outcome variables with three different categories. Time spent on the computer was a predictor variable, and sex and school grade (aged 12-13 and, 15-16 years) were considered potential confounders and treated as covariates. We calculated the odds ratios and 95% confidence intervals, and used P-values to show the differences between the age and sex groups. The statistical differences between the groups were tested using the chi-square test. A p-value of less than 0.05 was considered statistically significant.

## Results

Severe pain intensity and severe inconvenience to everyday life were reported most commonly for the head, neck-shoulders, and eyes, and least commonly for the hands, fingers, and/or wrists. The prevalence of moderate/severe pain was 20.7% for neck-shoulders, 19.7% for head, and 13.8% for eyes. The prevalence of moderate/severe inconvenience to everyday life was 28.3% due to head pain, 20.7% due to neck-shoulder pain, and 15.4% due to low back (Table 1). Pain intensity was reported as follows: girls reported more moderate/severe computer-associated pain than boys at all anatomic sites, except the low back, for which the prevalence was higher among the boys. Pain intensity in the neck-shoulders (p = 0.0001) and head (p = 0.0001) differed significantly between the sexes. Pain intensity at all anatomic sites increased with age, except in the eyes, for which the pain intensity decreased with age. The results were statistically significant in the neck-shoulders (p = 0.0001) and head (p = 0.0001) within age groups among both sexes. Level of inconvenience to everyday life was reported as follows: girls reported more moderate/severe inconvenience to everyday life caused by musculoskeletal pain than boys; head (p = 0.0001), neck-shoulders (p = 0.0001), and low back (p = 0.034) were the most prevalent sites for pain causing moderate/severe inconvenience. The 9^th ^grade pupils reported moderate/severe inconvenience to everyday life more often than the 6^th ^graders, and the results were statistically significant for head (p = 0.011), neck-shoulders (p = 0.0001), and low back (p = 0.0001) pain (data not shown).

**Table 1:** Level of inconvenience to everyday life caused by musculoskeletal pain, and intensity of computer-associated musculoskeletal pain, by sex and grade (%)

Symptoms	Boys	Girls			Total
	**6^th ^grade**	**9^th ^grade**	**6^th ^grade**	**9^th ^grade**	

	**(70)**	**(128)**	**(90)**	**(135)**	**(423)**

**Neck-shoulder**					

Level of inconvenience to everyday life					

Severe	1.4	2.4	5.6	19.3	7.2

Moderate	8.6	12.6	8.9	23.7	13.5

Mild	30.0	25.2	33.3	27.4	29.0

Pain intensity					

Severe	4.3	3.1	6.7	15.6	7.4

Moderate	10.0	11.8	10.0	21.5	13.3

Mild	22.9	26.0	33.3	24.4	26.7

**Low back**					

Level of inconvenience to everyday life					

Severe	0.0	4.7	1.1	11.1	4.2

Moderate	7.4	13.3	5.7	18.5	11.2

Mild	11.8	23.4	21.6	26.7	20.9

Pain intensity					

Severe	2.9	5.5	1.1	5.9	3.9

Moderate	7.1	12.6	5.6	12.6	9.5

Mild	12.9	19.7	23.6	22.2	19.6

**Head**					

Level of inconvenience to everyday life					

Severe	5.8	5.5	7.8	25.4	11.1

Moderate	15.9	10.9	14.4	27.6	17.2

Mild	30.4	32.8	34.4	29.1	31.7

Pain intensity					

Severe	7.2	3.9	3.4	15.7	7.6

Moderate	7.2	9.4	14.6	17.2	12.1

Mild	17.4	21.9	28.1	22.4	22.5

**Eyes**					

Level of inconvenience to everyday life					

Severe	4.3	1.6	4.4	8.9	4.8

Moderate	5.8	8.7	3.3	7.4	6.3

Mild	15.9	18.1	14.4	17.0	16.4

Pain intensity					

Severe	5.8	3.9	3.4	5.9	4.8

Moderate	8.7	7.8	11.2	8.1	9.0

Mild	13.0	23.4	25.8	26.7	22.2

**Hands, fingers, wrists**					

Level of inconvenience to everyday life					

Severe	0.0	1.6	3.4	6.0	2.8

Moderate	1.5	4.7	1.1	6.7	3.5

Mild	16.4	18.9	19.1	14.9	17.3

Pain intensity					

Severe	1.4	1.6	1.1	5.2	2.3

Moderate	2.9	3.1	4.5	7.4	4.5

Mild	18.8	11.0	21.3	14.1	16.3

A third (35.4%) of the respondents reported that they spend 14 or more hours a week using computers. Boys used computers more often than girls (p = 0.0001); half of the boys (50%, N = 98) and almost a quarter of the girls (23%, N = 51) used computers for 14 hours or more per week, which corresponds to 2 hours or more of daily use. The time spent using computers increased with age (p = 0.0001) among both sexes.

In the multinomial regression analysis, moderate/severe computer-associated musculoskeletal pain was significantly related to all anatomic sites when computer use was 14 hours or more hours/week. Moderate/severe pain in the neck-shoulders and head were statistically significantly related to computer use 3.6 to 13.99 hours/week. Moderate/severe inconvenience to everyday life due to musculoskeletal pain in the lower back and head was significantly related to computer use of 14 or more hours/week; and head pain was significantly related to computer use of 3.6 to 13.99 hours/week (Table 2).

**Table 2:** Odds ratios (OR) and 95% confidence intervals (Cl) for multivariate associations between computer use/week and level of inconvenience to everyday life caused by musculoskeletal pain, and intensity of computer-associated musculoskeletal pain.

Symptoms	Computer use time/week		
	**< 3.6 hours**	**3.6 - 13.99 hours**	**≥ 14 hours**

	n* OR**(95%Cl)	**n OR (95%Cl)**	**n OR (95%Cl)**

**Neck or shoulder**			

Level of inconvenience to everyday life			

Severe/moderate	27 1.0	40 1.3 (0.7-2.5)	31 1.1 (0.5-2.2)

Mild	29 1.0	50 1.3 (0.7-2.3)	39 1.1 (0.6-2.1)

Pain intensity			

Severe/moderate	17 1.0	42 **2.6 (1.3-5.3)**	35 **2.9 (1.4-6.1)**

Mild	26 1.0	44 1.6 (0.9-2.9)	41 1.9 (1.0-3.6)

**Low back**			

Level of inconvenience to everyday life			

Severe/moderate	15 1.0	26 1.5 (0.7-3.2)	34 **2.5 (1.2-5.5)**

Mild	18 1.0	39 1.8 (0.9-3.5)	33 **2.1 (1.0-4.3)**

Pain intensity			

Severe/moderate	9 1.0	19 1.6 (0.7-3.8)	32 **3.5 (1.5-8.3)**

Mild	14 1.0	37 **2.4 (1.2-4.8)**	34 **3.1 (1.5-6.7)**

**Head**			

Level of inconvenience to everyday life			

Severe/moderate	26 1.0	55 **2.0 (1.0-3.8)**	44 **2.0 (1.0-4.1)**

Mild	37 1.0	50 1.0 (0.6-1.9)	45 1.0 (0.5-1.9)

Pain intensity			

Severe/moderate	16 1.0	36 **2.4 (1.2-4.8)**	34 **3.4 (1.6-7.2)**

Mild	16 1.0	42 **2.6 (1.3-5.0)**	37 **3.0 (1.5-6.3)**

**Eyes**			

Level of inconvenience to everyday life			

Severe/moderate	12 1.0	16 1.1 (0.5-2.6)	21 1.9 (0.8-4.5)

Mild	12 1.0	27 1.6 (0.7-3.3)	30 **2.2 (1.0-4.8)**

Pain intensity			

Severe/moderate	11 1.0	22 1.8 (0.8-4.2)	25 **3.3 (1.4-7.8)**

Mild	16 1.0	38 **2.0 (1.0-3.9)**	44 **3.6 (1.8-7.3)**

**Hands, fingers or wrists**			

Level of inconvenience to everyday life			

Severe/moderate	7 1.0	10 1.5 (0.5-4.7)	14 2.7 (0.9-8.3)

Mild	15 1.0	31 1.4 (0.7-2.7)	24 1.2 (0.6-2.5)

Pain intensity			

Severe/moderate	4 1.0	12 2.3 (0.7-7.3)	15 **4.4 (1.3-14.5)**

Mild	18 1.0	21 0.9 (0.4-1.8)	27 1.7 (0.8-3.6)

Multinominal logistic regression analysis, adjusted for grade and sex

*n = Number of cases

**The reference category (no pain/no inconvenience) is indicated by an odds ratio (OR) of 1.0. Odds ratios are given in bold when they indicate a statistically significant difference from the odds of the reference category at 95% confidence level (CI).

Mild pain in the lower back, head, and eyes was significantly related to computer use of 14 or more hours/week and 3.6 to 13.99 hours/week. Mild inconvenience to everyday life was significantly associated with pain in the low back and eyes when computer use was 14 or more hours per week (Table 2).

## Discussion

The responses to two independent questions indicated that adolescents experience a low level of musculoskeletal pain with little effect on everyday life. On the other hand, our findings indicate that computer use of 14 or more hours/week (corresponding ≥ 2 hours/day) is related to moderate/severe computer-associated pain at all measured anatomic sites. Moreover, computer use of 14 or more hours/week was related to moderate/severe pain in the lower back and head, which affected the everyday lives of adolescents. Girls reported greater pain intensity and more inconvenience to everyday life due to pain at all anatomic sites than boys, and the prevalence rates increased with age.

To the best of our knowledge, this is the first study to evaluate pain intensity and level of perceived negative impact on everyday life related to computer use in adolescents. Our results suggest that computer use of 14 or more hours/week (estimated ≥ 2 hours/day) is related to severe or moderate pain. Computer use exceeding 2 hours/day is suggested to be a threshold for neck-shoulder pain [5] neck-shoulder pain is associated with 1 or more hours/day spent on the computer [11,23].

In our study, the pain intensity was lower than that reported in some previous studies [18,19,24], but approximately the same level as in the Finnish follow-up study by Saarni et al. with 88 participants [17]. Our results confirm earlier findings, that girls report more severe pain than do boys [18,19].

In the present study, the intensity of pain and inconvenience to everyday life were measured using the well-documented visual analogue scale, VAS. The VAS has been extensively studied and has shown good acceptability, responsivity, and validity for most children aged 8 years and older [25]. Before analysis of the data, pain intensity categories were defined based on the findings of Collins et al. [26]. Musculoskeletal symptoms in five anatomical locations were evaluated by two independent questions. These anatomical sites had been related to computer use in previous studies. The first question measured musculoskeletal symptoms in general with no reference to computer use. The second one measured musculoskeletal pain that children themselves attributed to their computer use. Using two different questions gives a more reliable picture and a wider perspective to the association between musculoskeletal symptoms and computer use. The questions and their VAS categories were stated in different sections of the questionnaire; computer-associated pain was stated in the ICT-section and musculoskeletal pain in the section concerning health status.

There are some limitations to the present study. The convenience sample of the present study does not necessarily represent the entire population of that age, although it is unlikely that this would influence the studied relationships. Due to the cross-sectional design of the study, we are restricted to investigating the contemporary relations in our data, and thus causal inferences cannot be made based on this study alone. As the questions used in the study measured the respondents' perception of the intensity of computer-associated pain, it was a subjective measure. Thus it was left to the respondents to report whether these symptoms were due specifically to computer use. Being a questionnaire survey, the rate of occurrence of computer -associated and musculoskeletal symptoms, the pain intensity and inconvenience to everyday life were based on self-reports, and therefore memory bias is possible and differences between individuals' interpretations cannot be ruled out [27]. Respondents reported the weekly duration of computer use in response to an open question. There may be misreporting and overestimation of the duration of adolescents' computer use especially if there are different procedures to measure computer time [10]. In adults, the use of self-reports can lead to the misclassification of computer exposure for more than 80% of respondents [28]. In a previous study of adults self-reported duration of computer use but not recorded computer exposure, was positively associated with upper extremity symptoms [29]. A study using a computer-based program showed that the use of a computer mouse predicted acute neck-shoulder pain, but not chronic neck-shoulder pain [30]. Overall, based on an overview of systematic reviews, computer work has been associated with upper extremity musculoskeletal symptoms, but the causal relationship between computer work and symptoms shows a more mixed level of evidence [31]. We also do not know either if the mechanisms by which computer use elicits pain differ between adults and adolescents. Future studies with longitudinal designs are needed to examine the persistence of musculoskeletal symptoms, with accurate measurements of computer use and to evaluate the changes between symptoms and computer use.

## Conclusion

The findings of the present study suggest that musculoskeletal symptoms causing moderate and severe pain as well as inconvenience to everyday life are common among adolescent computer users. Daily computer use of 2 hours or more increases the risk at most anatomic sites.

## Competing interests

The authors declare that they have no competing interests.

## Authors' contributions

AHR, R-LP, MAW, and C-HN initiated and designed the study, and LAS provided critical input in all phases. PTH and AHR performed the main analysis, drafted the paper, and coordinated subsequent revisions with the other authors. All authors read and approved the final manuscript.

## Pre-publication history

The pre-publication history for this paper can be accessed here:

http://www.biomedcentral.com/1471-2474/13/41/prepub
